# Profile of polio-compatible cases in Nigeria, 2006–2016

**DOI:** 10.1186/s12889-018-6184-0

**Published:** 2018-12-13

**Authors:** Abdullahi Walla Hamisu, Faisal Shuaib, Ticha Muluh Johnson, Kehinde Craig, Braka Fiona, Richard Banda, Sisay G. Tegegne, Ajiboye Oyetunji, Tesfaye B. Erbeto, Peter Nsubuga, Rui Gama Vaz, Ado J. G. Muhamed, Adamu Usman

**Affiliations:** 1 World Health Organization, Country Representative Office, Abuja, Nigeria; 2 National Primary Health Care Development Agency, Abuja, Nigeria; 3 Global Public Health Solutions, Atlanta, GA USA; 4 World Health Organization, Geneva, Switzerland

**Keywords:** AFP surveillance, Polio-compatible, Wild poliovirus

## Abstract

**Background:**

The tremendous progress made by Nigeria towards polio eradication has recently suffered a setback with the isolation of circulating vaccine-derived poliovirus (cVDPV) type 2 from environmental samples and confirmation of four wild poliovirus (WPV) cases from acute flaccid paralysis (AFP) cases, with dates of onset of paralysis in July and August 2016. All these viruses were confirmed from the security-challenged northeastern state of Borno. Polio-compatible cases exist in Nigeria, and they indicate surveillance failure. Surveillance, therefore, has to be strengthened for the country to achieve certification. The objective of this paper is to highlight the epidemiological profile and magnitude of polio-compatible cases in Nigeria during the reporting period, as well as immunization and surveillance response activities conducted to close immunity and surveillance gaps.

**Methods:**

We conducted a retrospective review of AFP surveillance performance and polio-compatible cases in Nigeria between 2006 and 2016 from the AFP database at the World Health Organization Country Office. We also reviewed and compared key epidemiological features of polio-compatible cases with those of wild poliovirus cases during the reporting period.

**Results:**

The non-polio AFP rate improved from 6.5 in 2006 to 19.5 in 2016. The corresponding figures for stool adequacy rates were 88 and 98%. The total number of polio-compatible cases reported during the reporting period was 888, with the highest number (194) of cases reported in 2006 and the least (24) in 2016. Clusters of polio-compatible cases were reported every year during the reporting period except in 2015. The highest number (65) of polio-compatible cases in clusters was reported in 2006. The key epidemiological features of polio-compatible and wild poliovirus cases were similar.

**Conclusion:**

AFP surveillance performance has improved significantly during the reporting period. Surveillance gaps still existed as shown by the presence of orphan viruses and polio-compatible cases, and these gaps need to be identified and closed to achieve certification.

## Background

The tremendous progress made by Nigeria towards polio eradication, especially in the last 2 years from 2014, suffered a setback in the second quarter of 2016. A circulating vaccine-derived poliovirus (cVDPV) type 2 was isolated from the environmental samples collected in March 2016 and four wild polioviruses (WPVs) from acute flaccid paralysis (AFP) cases with dates of onset of paralysis in July and August 2016 were confirmed. All these viruses were confirmed from the security-challenged northeastern state of Borno [1, 2].

For polio eradication, AFP surveillance is needed to identify possible areas of poliovirus transmission or cases of importation. Surveillance is also critical for documenting the absence of poliovirus circulation for polio-free certification [3]. A country’s surveillance system should be sensitive enough to detect at least one case of AFP for every 100,000 children under the age of 15 years, even in the absence of polio [4]. In the case of polio endemic countries, however, at least two cases of AFP for every 100,000 children under the age of 15 years are required [5].

In Nigeria, the AFP surveillance performance as gauged by the non-polio AFP and stool adequacy rates has been impressive during the period from 2006 to 2016 [6]. In 2016, for instance, the country recorded a non-polio AFP rate of 19.5 cases per 100,000 children < 15 years old (the target is 2.0/100,000). The stool adequacy rate during the same period was 98% (the target is 80%). Despite this performance, however, surveillance gaps, especially at subnational levels, exist as shown by the presence of polio-compatible cases.

Polio-compatible cases are those AFP cases for whom two adequate stool specimens were not collected, have negative laboratory results, 60-day follow-up reveals residual paralysis, and patients are lost to follow-up or died before follow-up. Such cases are referred to an expert committee which classifies them as compatible based on clinical, epidemiological, and laboratory evidence.

Additional evidence of surveillance gaps includes findings from rapid surveillance assessments conducted within the reporting period which showed knowledge gaps among key surveillance personnel, missed AFP cases, and inadequate active surveillance and documentation [7–10].

The occurrence of polio-compatible cases indicates surveillance failure and therefore the system may not be fully relied upon to exclude with certainty the existence of areas of poliovirus transmission. Similarly, such compatible cases should be monitored for clustering in space and time. Clustering occurs when two or more polio-compatible cases occur in any Local Government Area (LGA) with the date of onset of paralysis within 2 months of each other [11, 12].

Nigeria must strive to maintain a very sensitive polio surveillance system in order to achieve certification. All deficiencies in the surveillance system must be identified and addressed to ensure timely poliovirus detection and response. This was the path followed by other countries that have attained a polio-free status [13].

This study describes the performance of AFP surveillance and shows the key epidemiological features of polio-compatible cases and how these compare with those of WPV cases during the period between 2006 and 2016.

## Methods

### Study design, area, and population

We conducted a retrospective review of routinely collected data on AFP, WPV, and polio-compatible cases in Nigeria between 2006 and 2016.

### Description of the AFP surveillance system in Nigeria

The AFP surveillance system in Nigeria is both health facility- and community-based. There is a network of prioritized reporting sites (both public and private health facilities) and community informants comprising patent medicine vendors, traditional and spiritual healers, traditional bone setters, and traditional birth attendants spread across all the political wards, states, and LGAs in the country.

The health facility focal person in a reporting site, as well as other health workers or clinicians, detect and report AFP cases to the LGA Disease Surveillance and Notification Officer (DSNO). The DSNO, in turn, has the responsibility of investigating the reported cases including stool specimen collection and transportation to the national polio laboratory under reverse cold chain conditions. The LGA DSNO is supported by an assistant and World Health organization (WHO) LGA facilitator. Community informants refer AFP cases to the nearest reporting site or report cases directly to the LGA DSNO. Active surveillance at the LGA level is conducted by the LGA DSNO, their assistant, WHO LGA facilitators, and field volunteers.

At the state level, the state epidemiologist and the state DSNO coordinate surveillance activities including organizing monthly LGA DSNO meetings, outbreak investigation, supervision, training, and sensitization activities. The WHO cluster consultants support the state and LGA surveillance focal points, conduct active surveillance, verify reported AFP cases, and conduct 60-day follow-up of inadequate AFP cases, confirmed poliovirus cases, and cases with Sabin (i.e., vaccine) virus.

At the national level, surveillance is coordinated by the national epidemiologist who receives and analyses laboratory results from the two national polio laboratories and shares feedback with stakeholders, conducts supervision and monitors surveillance performance, and organizes surveillance assessments, outbreak investigation, peer reviews, and training, and supports the polio laboratories and the national polio committees. WHO supports the surveillance system at all levels including the provision of logistics support to the DSNOs, national polio laboratories, and the national polio committees.

There is a five-member National Polio Expert Committee (NPEC) which meets quarterly to classify AFP cases brought to it by the secretariat. The secretariat is provided by the National Primary Health Care Development Agency (NPHCDA) and supported by WHO.

### Data collection and analysis

We extracted data on the two core AFP surveillance performance indicators of non-polio AFP and stool adequacy rates. We specifically extracted data on age, sex, and doses of oral polio vaccine received for polio-compatible and WPV cases. We also identified the trend, number of states, and LGAs with clustering of polio-compatible cases.

## Results

**Fig. 1:**
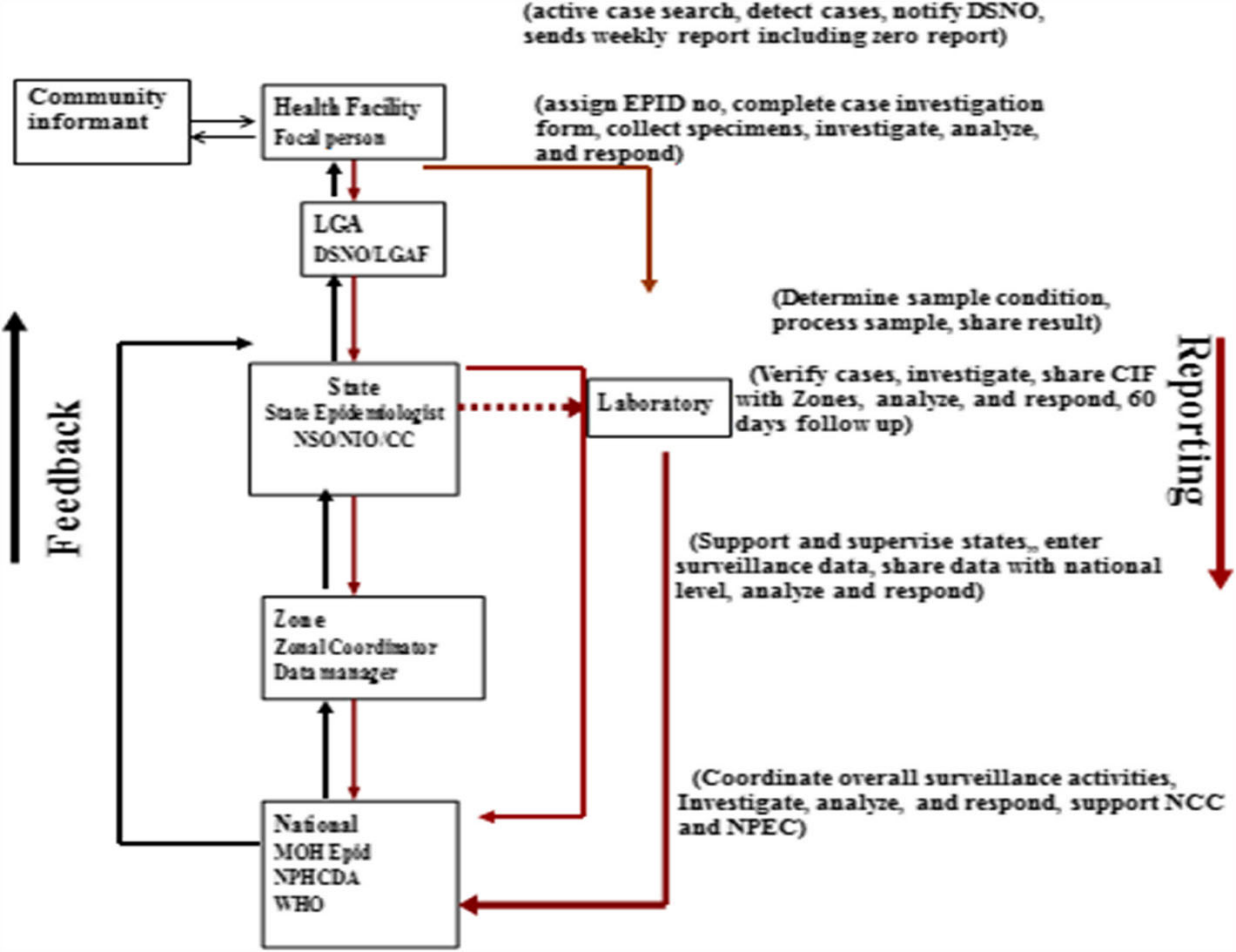
Polio surveillance system in Nigeria, 2016. Abbreviation: *CC* Cluster Consultant, *CIF* Case Investigation Form, *DSNO* Disease Surveillance and Notification Officer, Epidemiological, *LGA* Local Government Area, *LGAF* Local Government Area Facilitator, *MOH* Ministry of Health, *NCC* National Certification Committee, *NIO* National Immunization Officer, *NPEC* National Polio Expert Committee, *NPH CDA* National Primary Health Care Development Agency, *NSO* National Surveillance Officer, *WHO* World Health Organization

**Fig. 2:**
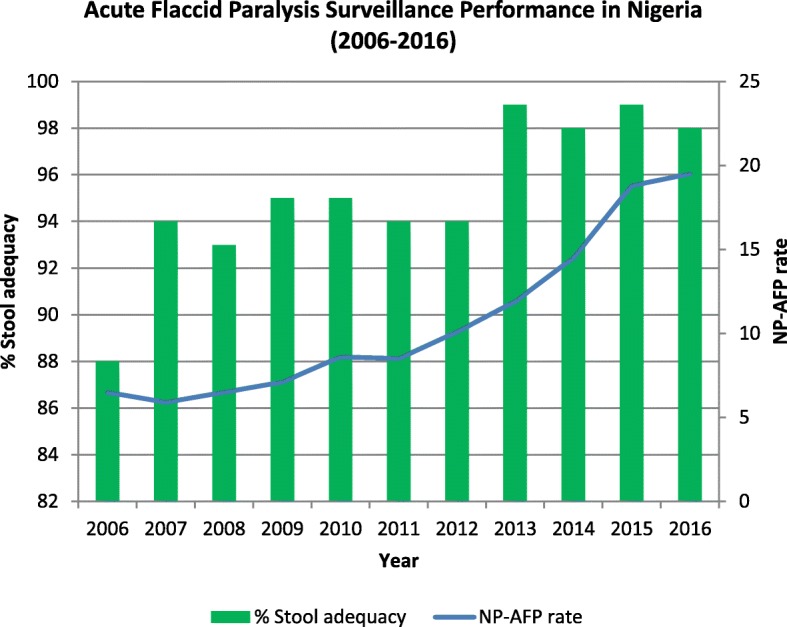
Acute flaccid paralysis surveillance performance in Nigeria (2006–2016). NP-AFP non-polio acute flaccid paralysis

The surveillance system in Nigeria is both health facility and community-based (Fig. 1). The non-polio AFP rate improved from 6.5 in 2006 to 19.5 in 2016. The corresponding figures for stool adequacy rates were 88 and 98% (Fig. 2).

During the reporting period, the total number of polio-compatible cases reported was 888 out of which 516 (58%) were males and 372 (42%) females. Most (82%) of the compatible cases were under the age of 5 years. A total of 137 (15%) of the compatible cases did not receive any oral polio vaccine (OPV), while 255 (29%) received 1–3 OPV doses, and 496 (56%) received 3+ OPV doses (Table 1).

**Table 1:** Trend and key epidemiological features of polio-compatible cases in Nigeria, 2006–2016

Year	Number of compatible cases	Sex		Age (months)				Oral polio vaccine doses		
		Male *n* (%)	Female *n* (%)	< 12 *n* (%)	12–35 *n* (%)	36–59 *n* (%)	60+ *n* (%)	0 *n* (%)	1–3 *n* (%)	3+ *n* (%)
2006	194	110 (57)	84 (43)	18 (9)	129 (67)	33 (17)	14 (7)	51 (26)	90 (46)	53 (28)
2007	74	41 (55)	33 (45)	5 (7)	46 (62)	13 (18)	10 (13)	22 (30)	31 (42)	21 (28)
2008	93	53 (57)	40 (43)	10 (11)	52 (56)	19 (20)	12 (13)	21 (23)	36 (39)	36 (38)
2009	75	41 (55)	34 (45)	4 (5)	40 (53)	13 (18)	18 (24)	9 (12)	23 (31)	43 (57)
2010	79	44 (56)	35 (44)	8 (10)	36 (46)	20 (25)	15 (19)	9 (11)	27 (34)	43 (55)
2011	141	93 (66)	48 (34)	8 (6)	59 (42)	42 (30)	32 (22)	14 (10)	25 (18)	102 (72)
2012	67	38 (57)	29 (43)	3 (4)	32 (48)	16 (24)	16 (24)	5 (7)	12 (18)	50 (75)
2013	81	45 (56)	36 (44)	5 (6)	36 (45)	13 (16)	27 (33)	5 (6)	7 (9)	69 (85)
2014	35	20 (57)	15 (43)	3 (9)	19 (54)	7 (20)	6 (17)	1 (3)	1 (3)	33 (94)
2015	25	16 (63)	9 (37)	3 (12)	16 (67)	2 (9)	3 (12)	0 (0)	3 (8)	22 (92)
2016	24	15 (63)	9 (37)	3 (13)	13 (54)	5 (12)	3 (12)	0 (0)	0 (0)	24 (100)
Overall	888	516 (58)	372 (42)	70 (8)	465 (54)	182 (20)	156 (18)	137 (15)	255 (29)	496 (56)

During the reporting period, there was a cluster of 235 polio-compatible cases. The highest number of states affected was in 2006 and 2011 with 11 and 10 states, respectively. The highest number of clusters (65 and 45) were also recorded in 2006 and 2011, respectively, and the corresponding number of affected LGAs were 26 and 19 in the same years (Table 2).

**Table 2:** Trend and distribution of compatible clusters in states and Local Government Areas (LGAs) in Nigeria, 2006–2016

	Compatible clusters		
Year	Number of compatible cases	Number of states	Number of LGAs
2006	65	11	26
2007	21	3	9
2008	28	7	11
2009	14	5	6
2010	22	7	10
2011	45	10	19
2012	17	6	8
2013	16	5	6
2014	4	2	2
2015	0	0	0
2016	3	1	1
Overall	235	56	97

Of the 2861 WPV cases reported during the reporting period, 1638 (57%) were males and 1223 (43%) females. A total of 843 (30%) of WPV cases did not receive OPV, while 1438 (50%) received 1–3 OPV doses, and 576 (20%) received 3+ OPV doses (Table 3).

**Table 3:** Trend and key epidemiological features of wild poliovirus (WPV) cases in Nigeria, 2006–2016

Year	Number of WPVs	Sex		Age (months)				Oral polio vaccine doses		
		Male *n* (%)	Female *n* (%)	< 12 *n* (%)	12–35 *n* (%)	36–59 *n* (%)	60+ *n* (%)	0 *n* (%)	1–3 *n* (%)	3+ *n* (%)
2006	1122	649 (58)	473 (42)	82 (7)	767 (68)	219 (20)	54 (5)	412 (37)	558 (50)	152 (14)
2007	286	163 (57)	123 (43)	24 (8)	183 (64)	66 (23)	13 (5)	73 (26)	155 (54)	58 (20)
2008	796	452 (57)	344 (43)	43 (5)	544 (68)	181 (23)	28 (4)	235 (30)	428 (54)	133 (17)
2009	388	225 (58)	163 (42)	25 (6)	237 (61)	94 (24)	32 (8)	66 (17)	197 (51)	125 (32)
2010	21	17 (81)	4 (19)	0 (0)	15 (71)	4 (19)	2 (10)	3 (14)	7 (33)	11 (52)
2011	63	40 (63)	23 (37)	3 (5)	39 (62)	14 (22)	7 (11)	20 (32)	22 (35)	21 (33)
2012	122	64 (52)	58 (48)	11 (9)	75 (61)	29 (24)	7 (6)	27 (22)	44 (36)	51 (42)
2013	53	24 (45)	29 (55)	4 (8)	30 (57)	12 (23)	7 (13)	7 (13)	21 (40)	25 (47)
2014	6	3 (50)	3 (50)	0 (0)	4 (67)	1 (17)	1 (17)	0 (0)	6 (100)	0 (0)
2015	0	0 (0)	0 (0)	0 (0)	0 (0)	0 (0)	0 (0)	0 (0)	0 (0)	0 (0)
2016	4	1 (25)	3 (75)	0 (0)	3 (75)	1 (25)	0 (0)	0 (0)	0 (0)	0 (0)
Overall	2861	1638 (57)	1223 (43)	192 (7)	1897 (66)	621 (22)	151 (5)	843 (30)	1438 (50)	576 (20)

## Discussion

We found that the AFP surveillance performance in the country has consistently improved from 2006 to 2016 with the highest level of performance in 2016 which was above the minimum requirement of WHO in each of those years [14]. The presence of polio-compatible cases, however, indicates the existence of surveillance gaps through failure to collect adequate stool specimens, and this is more so if there is clustering of compatible cases [15].

In Nigeria, clustering of polio-compatible cases occurred in each of the years during the reporting period, except in 2015. The country witnessed the highest number of clustering of compatible cases in 2006 and 2011, representing periods with particularly serious surveillance gaps and, despite the high number of WPV cases reported during these years, many more cases could have been missed as a result of failure to timely detect AFP cases. However, the consistent decline in the number of polio-compatible cases from 194 in 2006 to 24 in 2016 as well as the declining clustering of cases shows much-improved surveillance in the country. Surveillance gaps at this stage of polio eradication are a global threat and must be adequately addressed [16]. The occurrence of polio-compatible cases is a global phenomenon [17], and cases have been known to occur in countries with sensitive AFP surveillance [18–20].

The AFP surveillance system in Nigeria has a definite protocol for responding to polio- compatible cases. This response has both immunization and surveillance components. During field investigation of a compatible case, OPV is administered to those under 5 years of age in the vicinity (or settlement) of the case to serve as an immediate immunization response. The main immunization response, however, is that three rounds of immunization campaigns should be conducted with bivalent OPV (bOPV) targeting the age group of 0–59 months in the index ward with contiguous concentric wards depending on risk analysis using several datasets (routine immunization, supplemental immunization activities, mop up, and surveillance). The immunization response follows the following guide: once a case is classified as compatible, the number of supplemental immunization activities in the area since the date of onset of paralysis is determined. If supplemental immunization activities were conducted less than 1 month from onset of paralysis, then that immunization activity is considered to be a round-one response. Two immunization response activities should then be conducted 2 to 3 weeks apart. If no supplemental immunization activity was conducted less than 1 month from onset of paralysis, then three rounds of immunization activities will be conducted. If three or more rounds of immunization activities have been conducted from the date of onset of paralysis to the date of classification of the case in the locality, then there is no need for any immunization response.

For the surveillance response upon notification of a compatible case, the outbreak investigation team will conduct an investigation within 48 h, including a search for active cases in the affected community and in all health facilities in the affected ward and neighboring wards within 1 week of case notification. During the mop-up immunization activities, the DSNO provides training to vaccinators on case finding and reporting. In the Borno state, which is experiencing security challenges due to the insurgent Boko Haram, extra measures were taken to ensure quality immunization and surveillance responses through partnership with the military and civilian security organizations. Security personnel accompany vaccination teams during immunization campaigns in inaccessible and partially accessible areas and also support investigation of AFP cases. In addition, special teams were formed to reach high-risk populations in the Internally Displaced Persons’ camps and host communities.

Polio-compatible cases are not true polio cases but are classified as such since the occurrence of polio in such cases cannot be ruled out [21]. The occurrence of polio-compatible cases is, however, not inevitable since the final classification is largely dependent on the information available to the NPEC. Many countries, including Algeria and Rwanda, did not report any polio-compatible case during the reporting period [22]. Polio-compatible cases should be investigated to identify the underlying cause for effective intervention [23]. In addition to showing areas of surveillance gaps, compatible polio cases also influence the choice of areas to be targeted for mop-up campaigns [24].

The age distribution and sex ratio as well as geographic spread of polio-compatible and WPV cases were similar to the cases in the Democratic Republic of the Congo [25]. Male preponderance has, however, been noted in both wild and polio-compatible data during the study period as was noted in earlier studies, and this may be due to more parental concern about their male children [26].

We found that most (> 90%) inadequate AFP cases were due to late detection. Late detection and notification are often due to ignorance and poor perception of disease epidemiology by caregivers who seek alternative health care as their first choice because of the belief that paralysis symptoms are spiritually induced [27].

One of the key limitations of this paper is that data on the OPV received by both polio-compatible and WPV cases were historical and not based on records such as immunization cards. Such historical data may be subject to bias, including that of recall. This limitation may have accounted for the significant difference in the proportion of OPV “zero dose” in the two groups. Also, OPV doses received during routine and supplemental immunization activities were not differentiated, making it difficult to comment on the strength or otherwise of these forms of immunization strategies. Another limitation is the incomplete records of the detailed investigation of compatible and WPV cases to enable comparison of other epidemiological features such as environmental settings, socioeconomic status of caregivers, and travel history.

Delayed AFP detection may lead to late polio outbreak detection and response which, in turn, hinders progress towards eradication and substantially raises the cost of the eradication program [28]. Furthermore, late outbreak detection has led to re-established transmission in many hitherto polio-free countries [29].

## Conclusion

We conclude that AFP surveillance performance in the country has improved significantly during the reporting period. Surveillance gaps, however, still exist as evidenced by the presence of polio-compatible cases, and these gaps need to be identified and closed to achieve certification.

We recommend that strategies leading to early AFP detection such as the conduct of active surveillance, training, and community and clinician sensitization, as well as the expansion of the reporting network should be given high priority.

## Abbreviations

AFP: Acute flaccid paralysis
bOPV: Bivalent oral polio vaccine
cVDPV: Circulating vaccine-derived poliovirus
DSNO: Disease Surveillance and Notification Officer
LGA: Local Government Area
NPEC: National Polio Expert Committee
NPHCDA: National Primary Health Care Development Agency
OPV: Oral polio vaccine
WHO: World Health Organization
WPV: Wild poliovirus
